# John De Haven White, M.D., D.D.S.

**Published:** 1896-01

**Authors:** 


### Obituary.

### JOHN DE HAVEN WHITE, M.D., D.D.S.

THE death of Dr. White took place at the Masonic Home, Philadelphia, December 25, 1895, in the eightieth year of his age.

It is with regret that the announcement of his decease came to us too late for an extended notice of his life-work in this number. This will appear in the February issue. He was a born leader of men, and occupied the most prominent position in dentistry in his day and generation.

